# Postoperative Unilateral Neck Irradiation for Oral Cancer: Risk Factors for Contralateral Neck Recurrence and Implications for Patient Selection

**DOI:** 10.7759/cureus.109048

**Published:** 2026-05-17

**Authors:** Hiroaki Wakiyama, Tadamasa Yoshitake, Keiji Matsumoto, Yuko Shirakawa, Osamu Hisano, Masanori Takaki, Hikaru Imafuku, Mioko Matsuo, Wataru Kumamaru, Shintaro Kawano, Kousei Ishigami

**Affiliations:** 1 Department of Clinical Radiology, Graduate School of Medical Sciences, Kyushu University, Fukuoka, JPN; 2 Department of Otorhinolaryngology, Graduate School of Medical Sciences, Kyushu University, Fukuoka, JPN; 3 Section of Oral and Maxillofacial Surgery, Division of Maxillofacial Diagnostic and Surgical Sciences, Faculty of Dental Science, Kyushu University, Fukuoka, JPN; 4 Section of Oral and Maxillofacial Oncology, Division of Maxillofacial Diagnostic and Surgical Sciences, Faculty of Dental Science, Kyushu University, Fukuoka, JPN

**Keywords:** contralateral neck recurrence, oral cancer, postoperative irradiation, radiotherapy, unilateral neck irradiation

## Abstract

Introduction: Postoperative irradiation is commonly used in patients with oral cancer who are at high risk of recurrence. However, the need for contralateral neck irradiation remains controversial. This study evaluated the efficacy of unilateral neck irradiation and identified risk factors for recurrence in the contralateral neck.

Methods: This retrospective study included 85 patients with oral cancer (27-88 years of age; median, 65 years) who underwent postoperative unilateral neck irradiation at a single institution between 2010 and 2023. Recurrence patterns and late adverse events were analyzed. The ipsilateral neck control rate (INCR), neck control rate (NCR), progression-free survival (PFS), and overall survival (OS) were estimated using the Kaplan-Meier method. The two-year contralateral neck control rate (CNCR) was evaluated using log-rank testing and Cox proportional hazards regression.

Results: The median duration of follow-up was 45 months. At two years, the rates of INCR, NCR, PFS, and OS were 85%, 77%, 69%, and 82%, respectively. Recurrence was observed in 31 (36.5%) patients, including ipsilateral recurrence in 15 (17.6%), contralateral recurrence in 11 (12.9%), and distant recurrence in 16 (18.8%), with 6 (7.1%) showing contralateral-only recurrence. No grade ≥3 late adverse events were observed. Five or more metastatic lymph nodes, ≥T2 tumors, and newly diagnosed status were significantly associated with poorer CNCR.

Conclusion: Unilateral neck irradiation may be an effective strategy for selected patients with oral cancer, particularly those with fewer metastatic lymph nodes, lower T stage, or recurrent cases.

## Introduction

Oral cancer is a prevalent form of head and neck malignancy and was the 18th most commonly diagnosed cancer worldwide in 2020 [1]. Oral squamous cell carcinoma accounts for 90% of all oral cancer cases. Surgery, radiotherapy (external beam radiation therapy or brachytherapy), and chemotherapeutic agents such as cisplatin, carboplatin, 5-fluorouracil, paclitaxel, and docetaxel can be used to treat oral cancer [2]. Postoperative irradiation is recommended for patients with a high risk of recurrence, such as those with positive resection margins, extracapsular invasion, multiple lymph node metastases, and vascular or perineural invasion after surgery for oral cancer. One important clinical question is whether irradiation to the contralateral neck is necessary in all cases. Unilateral neck irradiation has the advantage of reducing radiation exposure to organs at risk, including the salivary glands and swallowing-related structures, thereby potentially improving quality of life [3]. Several predictors of contralateral metastasis based on pathological findings after bilateral neck dissection for oral cancer have been reported, including tumor stage, ipsilateral nodal status, high histopathologic grade, and midline extension [4-6]. However, the applicability of these findings to patients receiving postoperative unilateral irradiation remains uncertain. Therefore, this study evaluated the efficacy of postoperative unilateral neck irradiation for oral cancer and investigated clinical risk factors for contralateral neck recurrence.

## Materials and methods

This was a retrospective study conducted at a single institution. This study was approved by the ethical review board of our institution (No. 24211) and was conducted according to the Japanese Ethical Guidelines for Medical and Biological Research Involving Human Subjects. The requirement for informed consent was waived. We retrospectively identified 85 patients with histologically confirmed squamous cell carcinoma of the oral cavity who underwent surgical treatment between 2010 and 2023, followed by postoperative unilateral neck irradiation at Kyushu University Hospital. Among these patients, 31 were newly diagnosed, and 54 were classified as having postoperative recurrence following surgical resection of oral cancer. In recurrent cases, rT0 was defined as recurrence limited to lymph nodes without evidence of a primary tumor. In our institution, postoperative radiotherapy to the ipsilateral neck is administered only in cases where the following conditions are met: the primary tumor does not extend to the midline, and contralateral neck lymph node metastasis is not suspected based on preoperative imaging studies.

External beam radiotherapy was delivered as three-dimensional conformal radiotherapy or intensity-modulated radiotherapy using a linear accelerator (TrueBeam or TrueBeam STx; Varian Medical Systems, Palo Alto, CA, USA) with 4-, 6-, or 10 MV photons. Radiotherapy was delivered at a dose of 40-50 Gy in 20-25 fractions to the unilateral neck, followed by boost irradiation in cases of positive margins or extracapsular invasion, with a total dose ranging from 49.4 to 71.2 Gy (median: 60 Gy). The clinical target volume (CTV) included the primary tumor bed and ipsilateral cervical lymph node regions at risk in newly diagnosed cases. In recurrent cases, the CTV was individualized based on the pattern of recurrence and prior treatment, and did not necessarily include the primary tumor bed in all patients. Prophylactic irradiation of the contralateral neck was not performed. Concurrent chemotherapy was administered in 70 (82.4%) patients (cisplatin, n=38; S-1, n=31; tegafur-uracil, n=1).

Recurrence patterns were also evaluated. Follow-up evaluations were generally performed every 3-6 months using physical examination and imaging studies, including CT and/or MRI, at the discretion of the treating physicians. Recurrence was diagnosed based on clinical and radiological findings, with pathological confirmation obtained when feasible. Late adverse events were evaluated according to the National Cancer Institute's Common Terminology Criteria for Adverse Events version 4.0 [7]. The ipsilateral neck control rate (INCR), neck control rate (NCR), progression-free survival (PFS), and overall survival (OS) were calculated from radiotherapy initiation. Furthermore, the two-year contralateral neck control rate (CNCR) was analyzed using the log-rank test along with various clinical factors, including age, sex, performance status (PS), newly diagnosed vs. recurrent disease, pT/rT stage, pN/rN stage, primary tumor subsite and distance from the midline, resection margins, number of lymph nodes, extracapsular invasion, dose, and chemotherapy status. Factors identified as significant by the log-rank test were subsequently assessed for independent prognostic significance using the Cox proportional hazards model. Statistical analyses were conducted using JMP® ver. 16 (SAS Institute, Cary, NC, USA) and GraphPad Prism version 10.4.1 (GraphPad Software, San Diego, CA, USA); p-values less than 0.05 were considered statistically significant.

## Results

The patient characteristics are summarized in Table 1. The patients included 54 men and 31 women of 27-88 years of age (median: 65 years). Thirty-one cases were newly diagnosed, and 54 were recurrent. The primary lesions included 51 lesions on the tongue, 11 on the buccal mucosa, six on the maxillary gingiva, 15 on the mandibular gingiva, one on the hard palate, and one on the floor of the mouth. The study included 51 pT/rT0, 3 pT/rT1, 10 pT/rT2, 4 pT3, and 17 pT4 cases, as no recurrent cases were classified as rT3 or rT4. For nodal classification, three pN0 (all newly diagnosed cases), six pN/rN1, 14 pN/rN2b, and 62 pN/rN3b cases were included. All patients underwent ipsilateral neck dissection. Sixty-eight patients were reported with negative primary tumor resection margins, and 17 with close or positive margins. Postoperative pathology revealed lymph node metastases ranging from 0 to 11 (median, 2) and extracapsular invasion in 62 patients. 

**Table 1:** Patient characteristics

Variable	n (%)
Age, median (range), years	65 (27-88)
Sex	
Male	54 (63.5%)
Female	31 (36.5%)
Disease status	
Newly diagnosed	31 (36.5%)
Recurrent	54 (63.5%)
Primary lesion	
Tongue	51 (60.0%)
Buccal mucosa	11 (12.9%)
Maxillary gingiva	6 (7.1%)
Mandibular gingiva	15 (17.6%)
Hard palate	1 (1.2%)
Floor of mouth	1 (1.2%)
pT/rT	
T0	51 (60.0%)
T1	3 (3.5%)
T2	10 (11.8%)
T3	4 (4.7%)
T4	17 (20.0%)
pN/rN	
N0	3 (3.5%)
N1	6 (7.1%)
N2b	14 (16.5%)
N3b	62 (72.9%)
Neck dissection	
Unilateral	85 (100%)
Primary tumor resection margins	
Negative	68 (80.0%)
Close or positive	17 (20.0%)
Number of lymph node metastases	
Median (range)	2 (0-11)
Extracapsular invasion	
Yes	62 (72.9%)
No	23 (27.1%)

**Figure 1:**
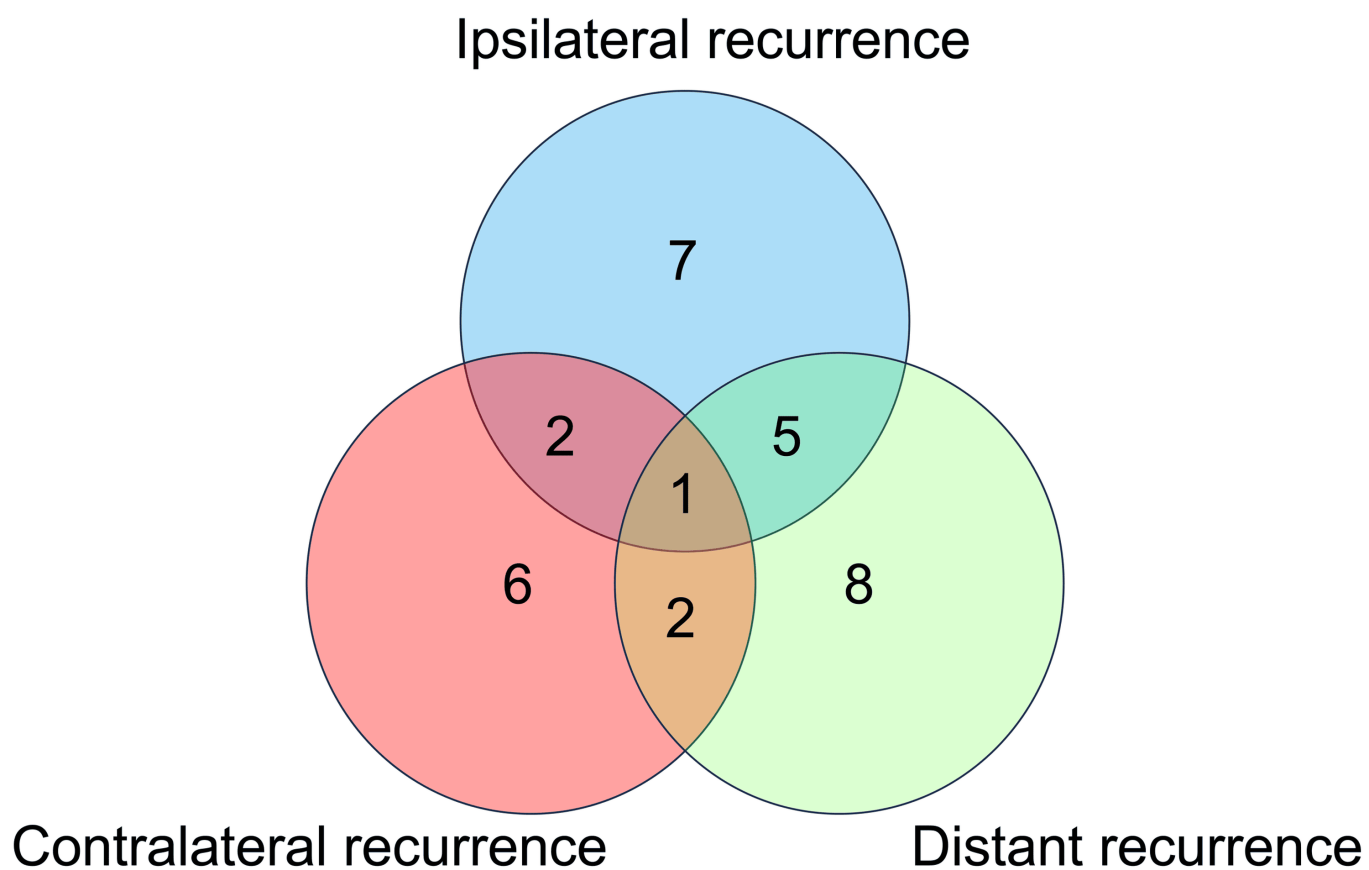
Pattern of recurrence. The Venn diagram shows the number of each recurrence pattern.

**Figure 2:**
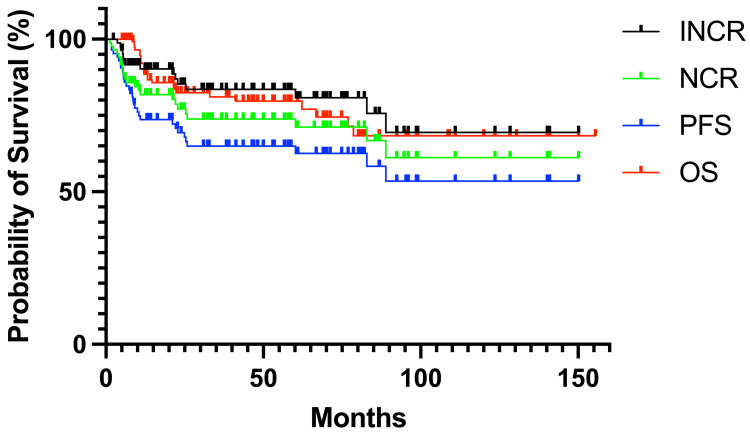
Kaplan-Meier survival curves for clinical outcomes. Kaplan-Meier survival curves of INCR (black line), NCR (green line), PFS (blue line), and OS (red line) of all patients. INCR, ipsilateral neck control rate; NCR, neck control rate; PFS, progression-free survival; OS, overall survival.

**Figure 3:**
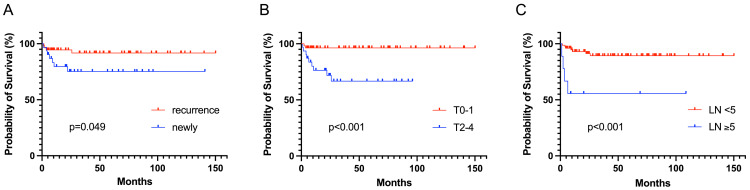
Kaplan-Meier curves for contralateral neck control. Kaplan-Meier survival curves for contralateral recurrence rate after postoperative radiotherapy divided by newly diagnosed or recurrent cases (A), T factor (B), and number of lymph node metastases (C). LN, lymph node.

The observation period was 5.2-156 months (median, 45 months). Eighteen deaths (21.2%) were reported, including 11 (12.9%) from the primary disease and 7 (8.2%) from other causes. No severe late adverse events of grade ≥3 were observed. Recurrence was observed in 31 (36.5%) patients, with ipsilateral recurrence in 15 (17.6%), contralateral recurrence in 11 (12.9%), distant recurrence in 16 (18.8%), and contralateral-only recurrence in 6 (7.1%) patients (Figure 1). The two-year INCR, NCR, PFS, and OS rates were 85%, 77%, 69%, and 82%, respectively. The five-year INCR, NCR, PFS, and OS rates were 84%, 74%, 65%, and 80%, respectively (Figure 2). The two-year contralateral recurrence rate was 4 (44.4%) in patients with ≥5 lymph node metastases versus 7 (9.2%) in those with <5; 8 (25.8%) in newly diagnosed patients versus 3 (5.6%) in recurrent cases; and 9 (29.0%) in patients with T2-4 tumors versus 2 (3.7%) in those with T0-1 tumors (Table 2, Figure 3).

**Table 2:** Clinical factors related to CNCR CNCR, contralateral neck control rate.

Factor	Category	n (%)	CNCR (2-year, %)	Univariate p-value	Multivariate p-value
Age	<65	42 (49.4%)	90%	0.297	-
	≥65	43 (50.6%)	85%		
Sex	Male	54 (63.5%)	90%	0.430	-
	Female	31 (36.5%)	82%		
Performance status	0-1	82 (96.5%)	88%	0.275	-
	2-3	3 (3.5%)	67%		
Recurrent case	No	31 (36.5%)	75%	0.049	0.029
	Yes	54 (63.5%)	94%		
Primary lesion	Tongue	51 (60.0%)	90%	0.227	-
	Other sites	34 (40.0%)	82%		
pT/rT	T0-1	54 (63.5%)	96%	<0.001	<0.001
	T2-4	31 (36.5%)	72%		
pN/rN	N0-1	9 (10.6%)	83%	0.835	-
	N2b-3b	76 (89.4%)	88%		
Primary tumor distance from midline	<5 mm	10 (11.8%)	79%	0.484	-
	≥5 mm	75 (88.2%)	88%		
Primary tumor resection margins	Negative	68 (80.0%)	87%	0.875	-
	Close or positive	17 (20.0%)	87%		
Number of lymph node metastases	≥5	9 (10.6%)	56%	<0.001	0.002
	<5	76 (89.4%)	91%		
Extracapsular invasion	No	23 (27.1%)	75%	0.163	-
	Yes	62 (72.9%)	92%		
Radiation dose	<60 Gy	15 (17.6%)	93%	0.403	-
	≥60 Gy	70 (82.4%)	86%		
Chemotherapy	Yes	70 (82.4%)	88%	0.945	-
	No	15 (17.6%)	85%		

## Discussion

We retrospectively evaluated 85 patients who underwent unilateral neck irradiation after surgery for oral squamous cell carcinoma. The number of metastatic lymph nodes, diagnostic status (newly diagnosed vs. recurrent), and tumor stage were identified as risk factors for the recurrence of contralateral neck metastases.

Kurita et al. reported that a higher number of ipsilateral lymph nodes is a risk factor for contralateral lymph node metastasis in oral cancer [6]. Another report indicated that the presence as well as the number of lymph node metastases correlated with an increased risk of contralateral neck recurrence after ipsilateral postoperative radiotherapy in patients with buccal mucosa squamous cell carcinoma [8]. A meta-analysis after unilateral neck irradiation for oral cancer reported that the isolated recurrence rate in the contralateral neck area was only 3.4%, and the clinical factor associated with recurrence in the contralateral neck area was the N factor [9]. The results of these studies are consistent with those of the present study, in which the risk of recurrence in contralateral neck lymph node metastases after ipsilateral radiotherapy increased when ≥5 ipsilateral lymph nodes were positive. Therefore, contralateral prophylactic irradiation should be considered for patients with multiple ipsilateral lymph node metastases.

A notable finding of this study is the difference between newly diagnosed and recurrent cases. Newly diagnosed patients showed a higher risk of contralateral recurrence, whereas recurrent cases had a relatively low risk. This may reflect differences in tumor biology and lymphatic spread patterns. In recurrent cases, the laterality of disease may already be established, reducing the likelihood of contralateral dissemination. Importantly, the inclusion of both newly diagnosed and recurrent cases represents a limitation of this study. However, this approach also enabled us to explore clinically relevant differences between these groups, which have not been well investigated in previous studies.

Tumor stage was also identified as a significant predictor of contralateral neck recurrence. A study including 66 patients with oral cancer in the N0-2 stage demonstrated contralateral occult metastasis rates of 8% for T2, 25% for T3, and 18% for T4 tumors, with no metastasis identified in T1 tumors [4]. Furthermore, bilateral metastases were rare in T1 cases [10] and are typically observed only in patients with T2 or more advanced disease [11]. The findings of these studies support the results of the present study, highlighting the significance of tumor stage in predicting recurrence in the contralateral neck area.

The advantage of unilateral neck irradiation over contralateral neck irradiation is that it reduces damage to at-risk organs such as the salivary glands and muscles of deglutition [3,12,13]. Bilateral neck irradiation has been reported to cause grade ≥2 dysphagia and xerostomia at six months post-treatment compared to unilateral neck irradiation [14]. In a study by Jensen et al. [15] with prospectively recorded toxicity outcomes comparing ipsilateral and bilateral neck irradiation groups, the rates of Grade 2 or greater xerostomia were 20% versus 61%, and Grade 2 or greater dysphagia were 10% versus 22%, respectively. Similarly, Liu et al. reported toxicity rates of 7% and 22% in the unilateral and bilateral neck irradiation groups, respectively [16]. Furthermore, unilateral neck irradiation results in shorter hospital stays, less weight loss, and less acute dysphagia compared to bilateral neck irradiation [17,18]. Thus, unilateral neck irradiation is generally less toxic than bilateral neck irradiation. In this study, patients with limited lymph node metastasis, low T stage, and recurrent disease showed a low risk of contralateral lymph node metastasis. Therefore, in such patients, unilateral neck irradiation can be actively considered to minimize late adverse effects and preserve quality of life (QoL). Notably, our findings also suggest that in cases of unilateral neck recurrence, contralateral irradiation may provide limited additional clinical benefit.

Several limitations should be acknowledged, including the retrospective design, the single-institution nature of the study, and the relatively small sample size. In addition, heterogeneity in treatment strategies may have introduced bias. In particular, surveillance intervals and treatment volumes in recurrent cases were individualized according to prior treatment history, recurrence patterns, and physician discretion, which may have introduced heterogeneity and potential selection bias into the analysis. Therefore, further prospective studies with standardized treatment protocols are warranted to validate these findings. In addition, caution is warranted when generalizing these findings to broader patient populations or institutions with different treatment strategies.

## Conclusions

Unilateral neck irradiation may be a safe and effective treatment strategy for selected patients with oral cancer. Patients with fewer metastatic lymph nodes, lower T stage, and recurrent disease may be appropriate candidates for this approach, while caution is warranted in newly diagnosed cases or those with high-risk features, particularly in patients with multiple lymph node metastases. These findings may help inform individualized treatment strategies and support decision-making regarding the extent of neck irradiation in patients with oral cancer. Careful patient selection based on clinical risk factors is essential to optimize oncologic outcomes while minimizing treatment-related toxicity. Further prospective studies with larger cohorts are warranted to validate these findings and to refine patient selection criteria for unilateral neck irradiation in clinical practice.
